# Homocystinuria symptomatology collected through an international patient-report data program as a source for natural history data

**DOI:** 10.1016/j.ymgmr.2026.101355

**Published:** 2026-09-11

**Authors:** Brittany Parke, Danae Bartke, Geoffrey Beek, Zohreh Talebizadeh, Kimberly A. Chapman, Joshua Baker, Janet A. Thomas

**Affiliations:** a HCU Network America, 15 S Mallory Ave, Batavia, IL 60510, United States of America; b Global Genes, 1012 14^th^ Street NW Suite 500, Washington, DC 20005, United States of America; c Children's Hospital Los Angeles, 4650 W Sunset Blvd, Los Angeles, CA 90027, United States of America; d Lurie Children's Hospital of Chicago, 225 E Chicago Ave, Chicago, IL 60611, United States of America; e University of Colorado School of Medicine, 13001 E 17th Pl, Aurora, CO 80045, United States of America

**Keywords:** Homocystinuria, Cobalamin disorders, MTHFR-deficiency, Patient reported data

## Abstract

Natural history data for homocystinuria is difficult to accurately obtain due to many factors related specifically to rare disorders. HCU Network America, a non-profit organization for this patient population, utilizes a patient-driven data collection program to support research. Patients and caregivers are asked to complete surveys through the collection program, and findings are provided as deidentified data. This report outlines the symptoms that enrolled participants identified as most impactful. Follow-up surveys were requested in those areas for specific symptomatology. Patients enrolled ranged from 1 to 74 years of age and encompass an international population from 23 countries and 35 US States.

## Highlights

• HCU Network America, a non-profit organization, created a patient reported data collection program powered by RARE-X to accelerate research.
• Patient reported data was queried to indicate the most common symptoms reported and compared based on diagnosis.
• This project supports that patient collected data is consistent with previously identified symptomatology for this patient population.
• It also highlights symptoms of other impactful patient features, such as bone abnormalities and headaches, often not discussed in historic literature.
• Patient reported data is a viable and valuable source of natural history data for researchers.

## 1 Introduction

Homocystinuria (HCU) is a group of rare, autosomal recessive disorders characterized by high levels of homocysteine in the blood and urine, often associated with thromboembolic and neurocognitive symptoms [1]. Classical homocystinuria is caused by impaired activity of the enzyme, cystathionine B-synthase (OMIM Ref.#613,381). Two phenotypes are prevalent and characterized as B6-responsive homocystinuria and B6-non-responsive homocystinuria with B6–responsive HCU usually presenting as a milder phenotype. Biochemical features of classical HCU are elevated plasma total homocysteine and methionine [2].

Elevated homocysteine levels can also occur due to inherited biochemical defects in cobalamin (cbl) processing and the remethylation pathway. This includes the following diagnosis: cblC, cblD, cblE, cblF, cblG, clbJ, clbK, clbX and MTHFR-deficiency (OMIM Ref# 609831, 611935, 602568, 612625, 156570, 603214, 603433, 300019, 607093, respectively). These disorders disrupt the cobalamin-dependent enzyme, methionine synthase, preventing the remethylation of homocysteine to methionine in one of three ways: 1. Impaired activity of the methionine synthase enzyme itself; 2. Impaired activity of an enzyme required to transport or generate the vitamin B12 cofactor, methylcobalamin; or 3. Impaired activity of the enzyme 5,10-methylene tetrahydrofolate reductase [1]. Biochemical features of remethylation defects vary from classical HCU in that patients present with normal or low levels of plasma methionine.

Early diagnosis and treatment are critical to prevent or reduce symptoms in patients. Although newborn screening provides early detection of many metabolic disorders, current newborn screening worldwide, with the exception of Qatar, use elevated methionine as the most common marker to identify classical HCU; screening does not target the cobalamin processing and remethylation defects and most are missed. Specifically, current screening lacks the ability to screen for isolated cobalamin defects, especially in patients without an elevated C3/C2 ratio. Additionally, individuals with classical HCU who are pyridoxine (vitamin B6) responsive are most often missed on newborn screening depending on individual state lab cutoffs for methionine, as methionine levels may not be elevated in the newborn period [3].

Treatment for homocystinuria varies based on the pathogenic variant and classification of the disorder, with the aim of lowering homocysteine levels [4], [5]. Data suggest that patients may be safe from serious complications with plasma total homocysteine levels up to 100 μmol/L. [4] More data is needed to determine if even lower levels of total homocysteine would lead to fewer disease manifestations and improve outcome without jeopardizing the quality of life of patients. Management for classical homocystinuria typically includes supplementation with betaine, pyridoxine, vitamin B12, and folic acid, along with a low protein or methionine restricted diet supplemented with a methionine free amino acid formula and use of low protein medical foods [4].

Cobalamin-related defects do not follow a low protein diet as it is contradictory due to already low methionine levels [6]. Betaine should be used to facilitate the remethylation of homocysteine to methionine. Depending on the specific remethylation or cobalamin-processing disorder, hydroxocobalamin may be used, and despite limited evidence, folinic acid, levocarnitine, and methionine may be added [5].

HCU Network America (HCUNA) is a non-profit 501(c)(3) organization that supports patients and families, provides education, funds research, and advocates for patients with homocystinuria. A 501(c)(3) organization is a type of nonprofit organization in the United States that is recognized by the Internal Revenue Service (IRS) as tax-exempt. To better serve the HCU community, HCUNA, in collaboration with RARE-X, a federated data analysis platform, conducted a survey to assess symptoms and findings reported by patients with homocystinuria (HCU) and their caregivers. The study included patients with cystathionine beta-synthase (CBS) deficiency, isolated remethylation defects (cblE, G and MTHFR-deficiency), and cobalamin processing disorders (cblC, D, F, J, K, X).

HCUNA's goal in creating the Homocystinuria Data Collection Program was to improve the natural history data of this disease group.

## 2 Methods and materials

HCU patients or caregivers of any age were invited through social media, patient newsletters, and during community meet ups for HCUNA and other patient organizations to participate in this ongoing, self-report study. There are currently 117 patients, with HCU, enrolled in RARE-X from 23 countries and 35 US states. Patient ages range from 1 to 74 years of age. Participation was voluntary, and all participants signed the online, electronic consent prior to filling out the questionnaires. Participation included completion of annual, standardized questionnaires provided by RARE-X [7]. RARE-X is the research program of Global Genes, a 501(c)(3) non-profit organization dedicated to eliminating the burdens and challenges of rare disease for patients, their families and disease communities globally. In partnership with rare disease communities, RARE-X enables data collection through its online platform to address the needs of patient communities and advance rare disease research. The platform's standardized questionnaires were designed to capture patient-reported information on symptoms, therapies, and quality of life for affected individuals. Participants were not excluded based on disease severity or vital status.

The RARE-X survey structure begins with a general health survey called “head-to-toe,” adapted from and expanded upon the GenomeConnect Registry developed by ClinGen to capture comprehensive baseline health information [8]. Survey assignments are then determined using branching logic based on participants' initial responses, followed by in-depth symptom area surveys called level 2 s (L2). Consequently, the number of completed second tier surveys (L2) varies greatly pending the choices on the “head-to-toe” survey. Governance and ethical oversight for the data collection platform are provided by RARE-X through the Univo Institutional Review Board (IRB). Participants or their caregivers complete an electronic consent or assent process tailored to age and cognitive ability, following an initial pre-screening step. The RARE-X platform does not collect treatment assessments and outcomes.

For this study, the database was queried for the most reported symptoms. If a patient had completed the same survey multiple times, only the most recent responses were included. Questionnaire answers from those with classical HCU and those with other forms of HCU were compared. Within the RARE-X platform, patients with isolated remethylation defects (cblE, cblG), cobalamin processing disorders (cblC, cblD, cblF, cblJ, cblK, cblX) and MTHFR-deficiency are referred to as ‘other homocystinuria’. Participant demographics are shown in Table 1.

**Table 1:** Demographic information of patients enrolled in RARE-X.

	Total Number of Patients	Patient Mean Age In Years	Patient Age Range (Yrs)	Patient Median Age (Yrs)	Standard Deviation of Patient Age	Male Patients	Female Patients	Survey completed by caregiver	Patient completed surveys	Located within USA	Located Outside USA
Classical HCU	84	25.9	1–74	21	18.98	26	58	37	47	49	35
Other HCU	33	15.7	1–56	6	15.84	16	17	25	8	23	10

### 2.1 Results

A total of 54 out of 84 patients, or their caregivers, with classical homocystinuria completed a head-to-toe symptom questionnaire. The results are summarized in Table 2. The most frequently reported symptom involvement was associated with vision (37/54), brain and nervous system (29/54), bones, cartilage, and connective tissue (27/54), digestive (26/54), and behavior or psychiatric (25/54). Twenty-six patients out of 33, or their caregivers, with other forms of HCU including those with cobalamin-processing disorders, remethylation defects and MTHFR-deficiency, also completed the same survey. The most reported challenges were in the brain and nervous system (21/26), developmental issues (18/26), vision (15/26), muscles (15/26), and digestive symptoms (14/26).

**Table 2:** Table of the symptoms comparing Classical Homocystinuria and Other Homocystinuria's (HCU). Number of each cohort with percentages in paratheses.

Table of Symptoms		
	Classical HCU (54)	Other HCU (26)
Vision	37 (69%)	15 (58%)
Brain/Nervous System	29 (54%)	21 (39%)
Digestive	26 (48%)	14 54%)
Bones/Cartilage/Connective Tissue	27 (50%)	–
Behavior/Psychiatric	25 (46%)	–
Development	–	18 (69%)
Muscles	–	15 (58%)

### 2.2 Classical homocystinuria

**Fig. 1:**
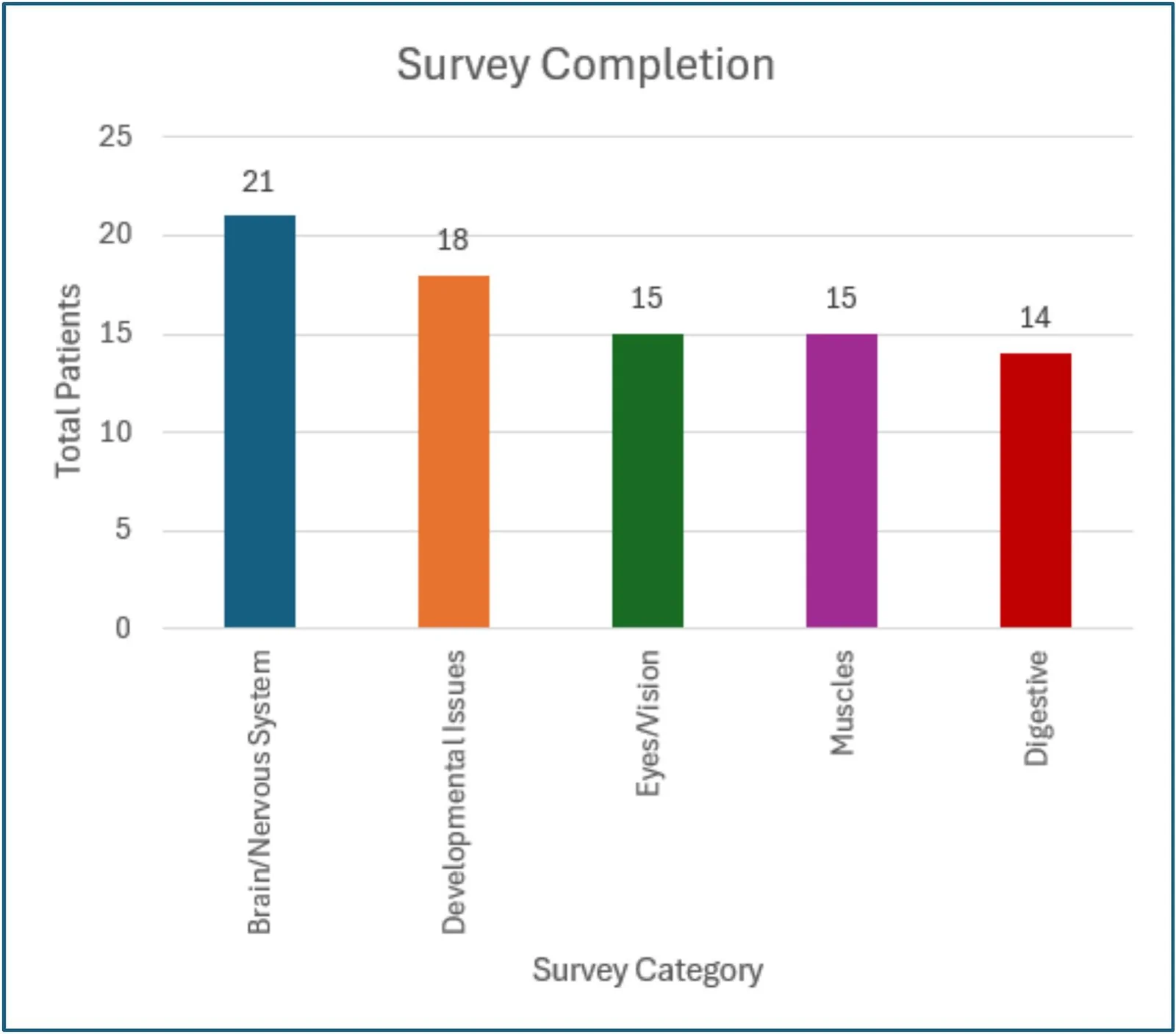
Total patients completing survey category.

**Fig. 2:**
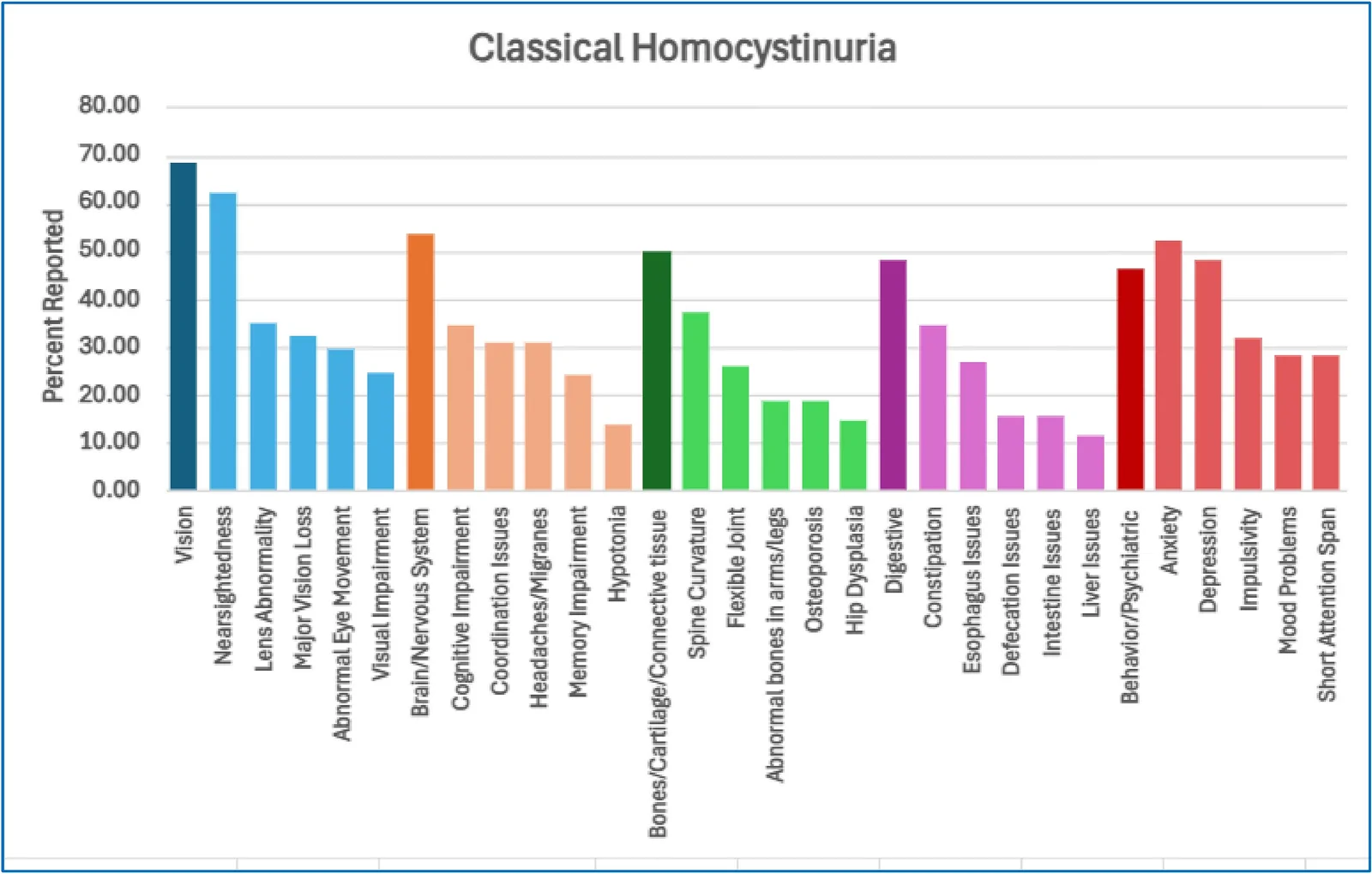
Top five reported areas and corresponding symptoms for Classical HCU shown in percentages. Main categories noted in dark color; subsection symptoms noted in light colors.

Following the completion of the *head-to-toe* symptom survey, enrolled participants were requested to complete follow-up surveys that provided more details about their noted symptom areas. Totals for survey completion are illustrated in Fig. 1. Fig. 2 illustrates the most frequent symptoms in classical HCU by category (main category noted in dark colors, symptoms noted in light colors). As noted, abnormalities in vision were the most common problem reported by 69% of the survey population. Patients were then prompted to complete the *L2 Vision survey* noting that nearsightedness (23/41) was the highest reported symptom followed by lens abnormality (13/41), major vision issues (12/41), abnormal eye movement (11/41), and visual impairment (9/41). The second major impacted category are findings consistent with impact on the brain and nervous system with patients reporting symptoms of cognitive impairment (10/43), coordination issues (9/43), headaches/migraines (9/43), hypotonia (4/43), and abnormal EEG (3/43) among others (reported on *L2 Brain and Nervous System survey*). Bones, cartilage and connective tissue issues were reported as spine curvature (10/44), flexible joints (7/44), abnormal bones in the arms/legs (5/44), osteoporosis (5/44) and hip dysplasia (4/44) through the *L2 Bones, Cartilage and Connective Tissue survey*. Next, 48% of patients were prompted to complete the *L2 Digestive survey* due to reported digestive issues, specifically constipation (9/43), issues with the esophagus (7/43), defecation (4/43), intestine (4/43), and liver (3/43). Finally, behavior was rated fifth at 46% with anxiety (13/45), depression (12/45), impulsivity (8/45), mood problems (7/45), and short attention span (7/45) comprising the top responses on the *L2 Behavior survey*. Other areas of concern not listed due to small prevalence were issues related to sleep, skin, growth, heart/blood vessels, teeth/mouth, muscles, development, heart, blood, and lungs.

### 2.3 Other homocystinuria

**Fig. 3:**
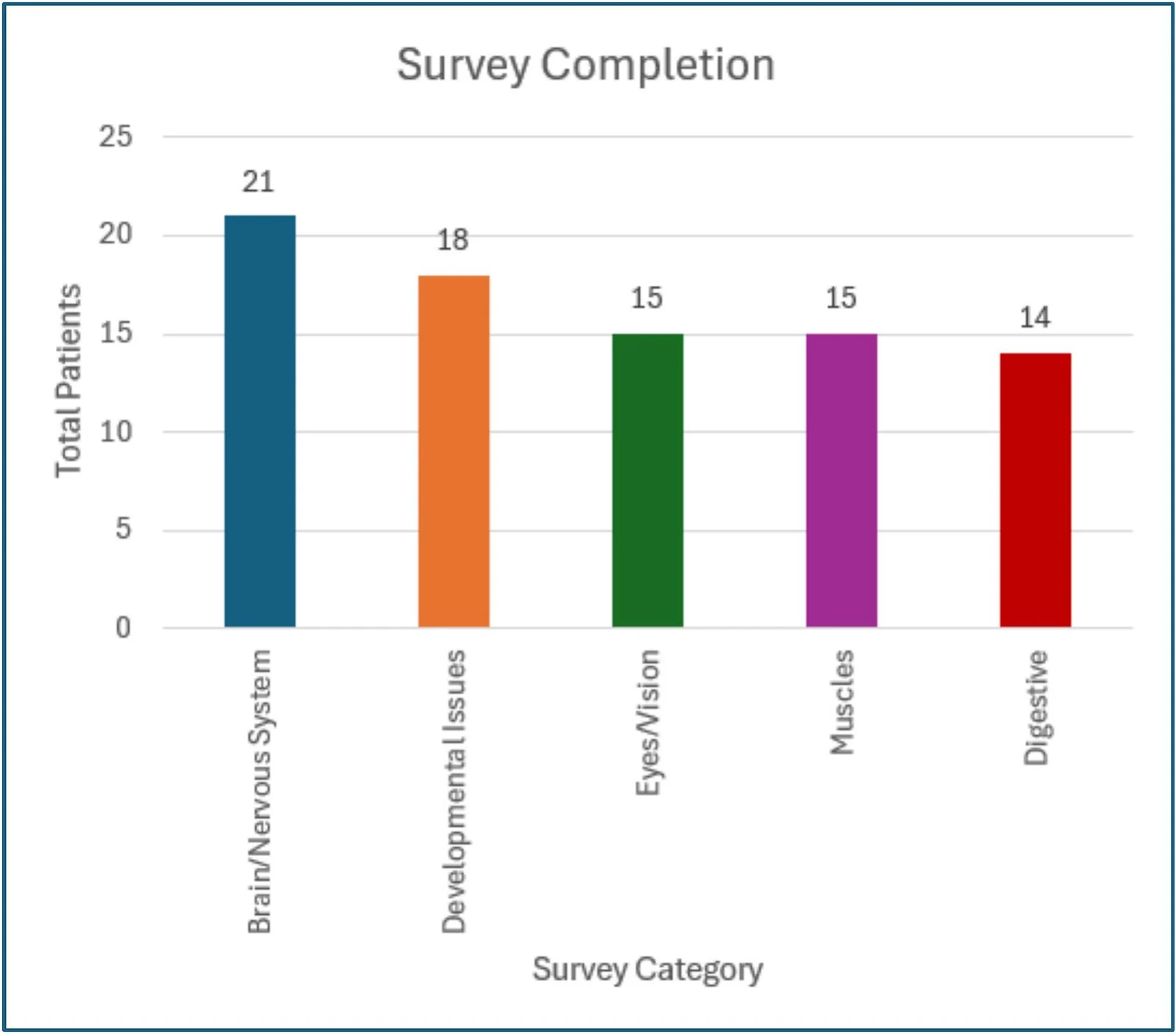
Total patients completing survey category.

**Fig. 4:**
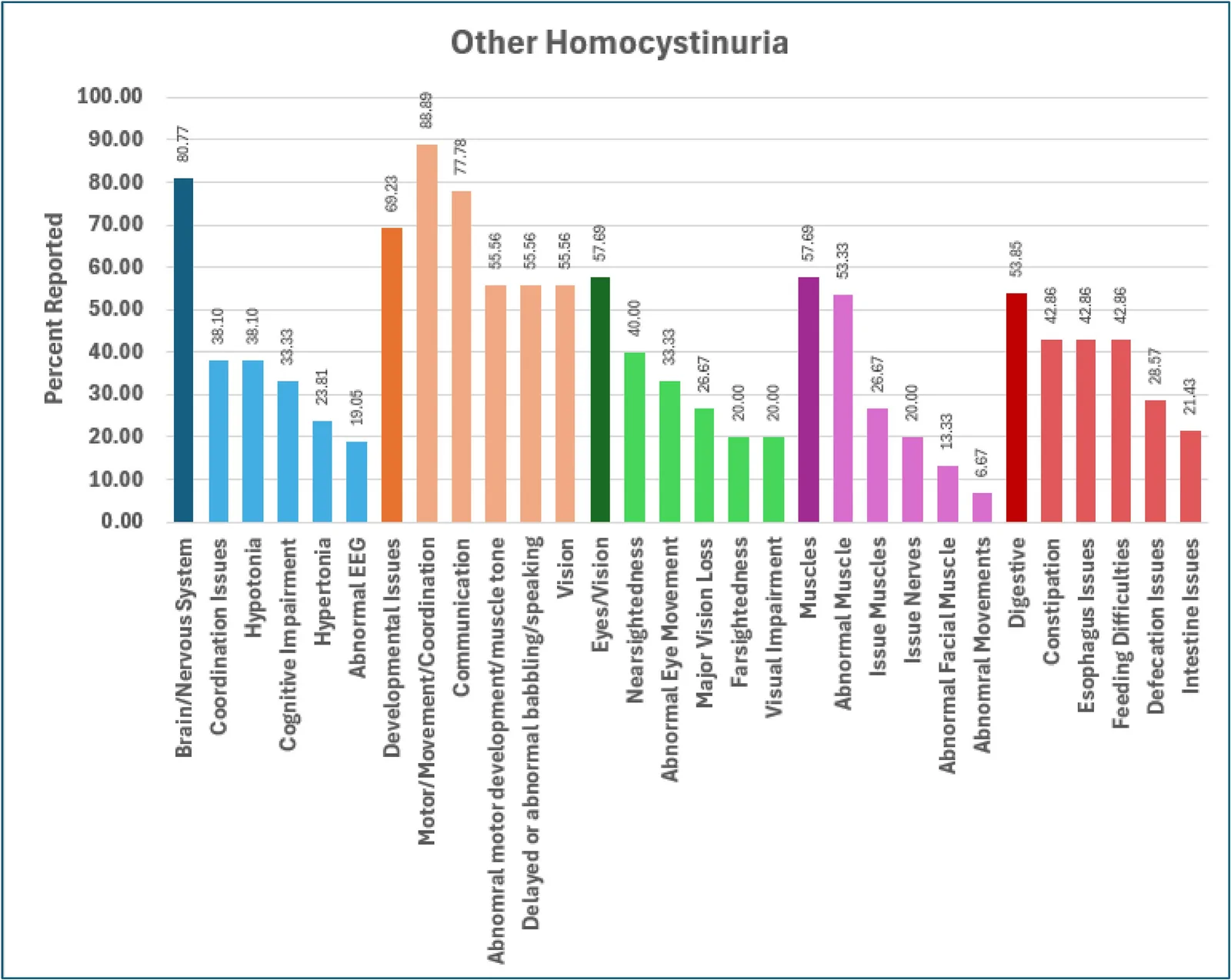
Top five reported areas and corresponding symptoms for other forms of HCU shown in percentages. Main categories noted in dark color; subsection symptoms noted in light colors.

Fig. 3 shows the total number of patients completing each survey category. Patients with other HCUs reported the most impacted area as the brain and nervous system, with 21 out of 26 patients (81%) highlighting these symptoms. These reported concerns then triggered the assignment of the *L2-Brain and Nervous System survey,* which patients and caregivers completed to provide more information about the impacted area. The specific symptoms reported were coordination issues (8/19), hypotonia (8/19), cognitive impairment (7/19), hypertonia (5/19), and abnormal EEG (4/19). The second most impacted area was development, with 18 patients (69%) reporting symptoms on *L2-The Health and Development survey.* The concerns were noted as motor/movement/coordination issues (16/26), communication (14/26), abnormal motor development or muscle tone (10/26), delayed or abnormal babbling or speaking (10/26), and vision (10/26). Vision concerns were noted on the *L2-Eye and Vision survey*, with 15 out of 26 patients (58%) reporting symptoms as nearsightedness (6/20), abnormal eye movements (5/20), major vision loss (4/20), farsightedness (3/20), and visual impairment (3/20). Fifteen patients reported symptoms related to muscles (58%) and completed the *L2*-*Muscles* survey. Abnormal muscle function (8/19), muscle issues in the arms and legs (4/19), nerve issues (3/19), abnormal facial muscles (2/19), and abnormal back muscles (1/19) were reported. The last area of concern was reported as digestion with 14 out of 26 patients (54%) reporting symptoms through the L2-*Digestive System* survey. Constipation (6/22), esophagus issues (6/22), feeding difficulty (6/22), defecation issues (4/22), and intestine/stomach issues (3/22) were the top reported symptoms. Fig. 4 depicts each category (in dark blue, dark orange, dark green, dark purple and dark red) and subsection reported (in light blue, light orange, light green, light purple and light red). Other areas of concern were noted in growth, sleep, loss of skills, behavior, bones/cartilage/connective tissues, heart/blood, skin, kidney/bladder and teeth/mouth; however, noted only in small numbers.

**Fig. 5:**
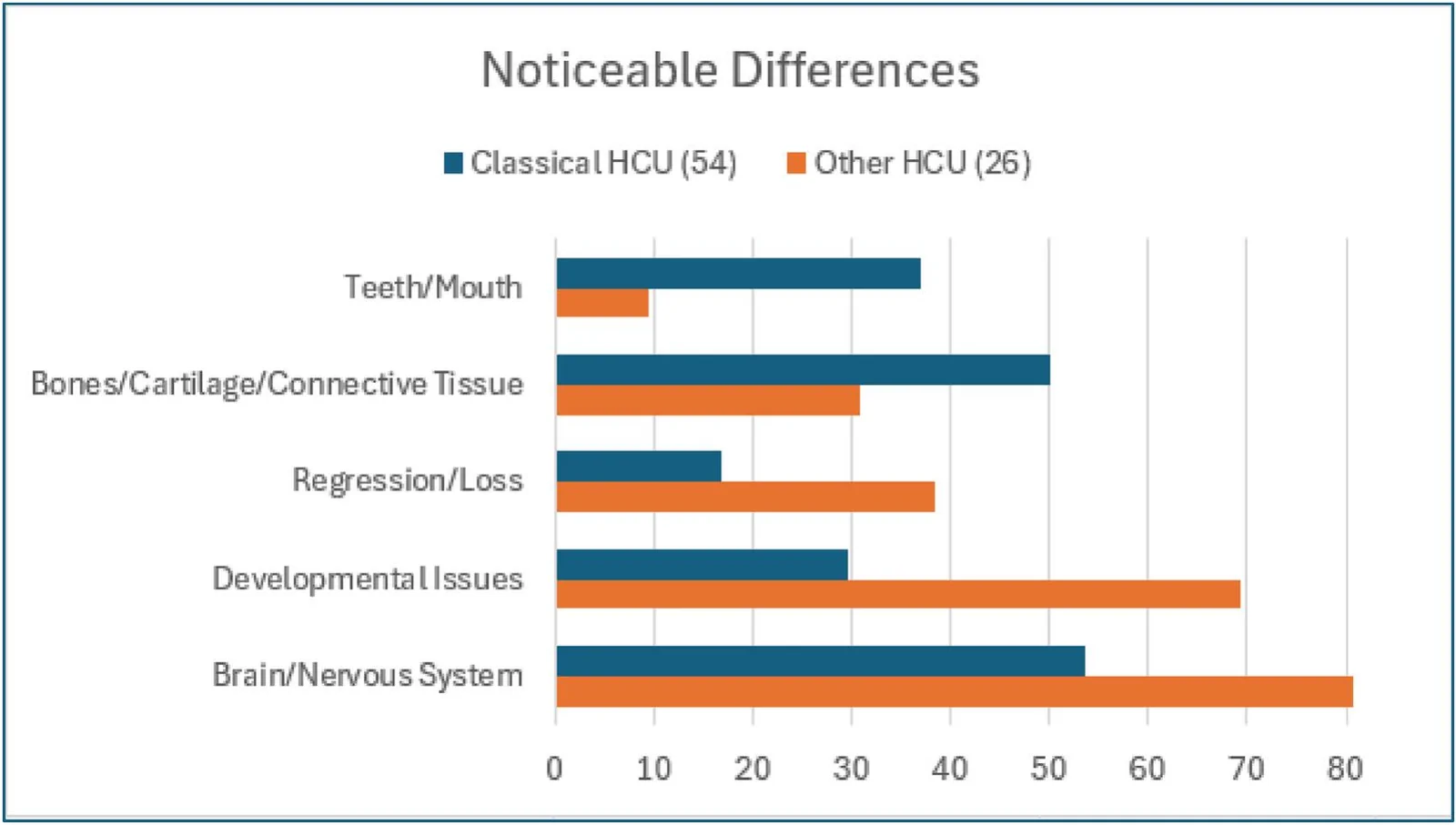
Noticeable differences in symptomology of classical HCU patient's vs other forms of HCU shown in percentages.

Fig. 5 illustrates the noticeable differences in symptomology between classical HCU and other forms of HCU. It was noted that symptoms were reported more frequently in other forms of HCU for brain/nervous system issues (+27%), developmental issues (+40%), and regression/loss of skills (+21%). In contrast, classical HCU patients reported higher incidences of bones/cartilage/connective tissue issues (+19%) and teeth/mouth issues (+27%) compared to other forms of HCU.

## 3 Discussion

HCUNA in collaboration with RARE-X sought to curate and gather information about the most common symptoms and signs of both classical HCU and other forms of HCU, including cobalamin-processing and remethylation defects.

Updated natural history data is long overdue for this patient population. Previous studies do not reflect the changes in diagnostic and management practices. Natural history studies of homocystinuria are challenging to fund and implement due to its rare prevalence. Patients are geographically dispersed and few in number. Patient driven data collection programs are an effective, accurate, convenient, and cost-effective tool to support documentation of the natural history of a disease using a patient center research approach. However, it should be noted that patients may associate symptoms with HCU that are true and true unrelated, especially as patients age. All features reported may not be related to HCU; certainly not causative [5]. While older natural history data exists for classical HCU, it is lacking for other HCUs, namely, remethylation disorders and MTHFR-deficiency, focusing mainly on single case studies and cobalamin C defects only.

The Homocystinuria Data Collection program provides opportunities for researchers to access de-identified patient data and accelerate research for all homocystinuria's. This project supports that patient collected data is consistent with previously identified symptomatology for this patient population and may highlight improvements in treatment and diagnosis. While classical HCU literature focuses on stroke and DVT symptomology, it was noted that neither of those symptoms were reported as high concerns for patients and caregivers. This could be due to better management of the disorder and treatment options resulting in these complications occurring less frequently, but further study would be needed to confirm this assumption.

Although treatment assessment and outcomes were not captured, the dataset documents patient-reported clinical features and symptom patterns. As such, it contributes to the understanding of disease natural history by describing the spectrum and frequency of manifestations within this population. The data provides cross-sectional and patient-reported information on symptom burden and clinical manifestations, representing a valuable source of natural history data that characterizes disease presentation independent of therapeutic intervention.

Data collected in this patient-reported research platform could be useful in expanding the clinical phenotype of HCU and similarly, in other rare disorders. As information is de-identified and aggregated, researchers may use this data for further research or to generate new research hypotheses. Unique or composite symptomology may provide insight to providers awaiting genetic confirmation of a diagnosis and allow early treatment to decrease serious complications.

Further, researchers and clinicians may use this patient-reported data to support the creation of clinical outcomes for future clinical trials. With better management and treatment options for patients, adjusting outcomes is imperative to the success of a clinical trial.

Subjectivity and bias may be a weakness in collecting data through a patient collection program. It is likely that the data is influenced by a bias of ascertainment with the patient population skewed to those individuals who feel more impacted by their diagnosis and hence, are more driven to participate in patient support organizations and associated projects. Limitations within the RARE-X platform such as language translation presents another weakness as it is currently only available in English. Further, patients are encouraged to complete all surveys and update answers yearly. However, only 69% of patients in the database have completed the main, head-to-toe survey. HCUNA recognizes these data weaknesses and continues to advocate for complete data collection by patients and caregivers in this ongoing natural history study.

## CRediT authorship contribution statement

**Brittany Parke:** Writing – review & editing, Writing – original draft, Project administration, Investigation. **Danae Bartke:** Writing – review & editing. **Geoffrey Beek:** Software, Data curation. **Zohreh Talebizadeh:** Data curation, Software. **Kimberly A. Chapman:** Writing – review & editing, Methodology, Conceptualization. **Joshua Baker:** Writing – review & editing. **Janet A. Thomas:** Writing – review & editing, Supervision.

## Funding sources

This research did not receive any specific grant from funding agencies in the public, commercial, or non-profit sectors.

## Declaration of competing interest

The authors declare the following financial interests/personal relationships which may be considered as potential competing interests: Brittany Parke reports a relationship with HCU Network America that includes: board membership and employment. If there are other authors, they declare that they have no known competing financial interests or personal relationships that could have appeared to influence the work reported in this paper.

Kimberly A Chapman, MD PhD reports a relationship with HCU network America that includes: board membership. If there are other authors, they declare that they have no known competing financial interests or personal relationships that could have appeared to influence the work reported in this paper.

Janet A. Thomas reports administrative support was provided by HCU Network America. Janet A. Thomas reports a relationship with HCU Network America that includes: board membership. If there are other authors, they declare that they have no known competing financial interests or personal relationships that could have appeared to influence the work reported in this paper.

Joshua Baker, DO reports financial support was provided by Travere Therapeutics Inc. Joshua Baker, DO reports financial support was provided by Eton Pharmaceuticals Inc. Joshua Baker, DO reports a relationship with HCU America that includes: board membership. If there are other authors, they declare that they have no known competing financial interests or personal relationships that could have appeared to influence the work reported in this paper.

## Data Availability

The Homocystinuria Data Collection Program is available for researchers at https://globalgenes.org/about-us/about-rare-x/.
